# Theoretical origin of genetically homologous *Plasmodium vivax* malarial recurrences

**DOI:** 10.4102/sajid.v37i1.369

**Published:** 2022-03-30

**Authors:** Miles B. Markus

**Affiliations:** 1 Wits Research Institute for Malaria, Faculty of Health Sciences, University of the Witwatersrand, Johannesburg, South Africa; 2 School of Animal, Plant and Environmental Sciences, Faculty of Science, University of the Witwatersrand, Johannesburg, South Africa

**Keywords:** epidemiology, genotyping, hypnozoite, identity by descent, meiotic sibling, *Plasmodium vivax*, primaquine, relapse, single-cell sequencing, whole-genome sequencing

## Abstract

Malaria caused by *Plasmodium vivax* is being diagnosed with increasing frequency in Africa. Some southern countries where it has been detected are Angola, Botswana, Mozambique, Namibia, Zambia and Zimbabwe. Knowing the parasite origin of *P. vivax* infection recurrences (which can be reinfections, recrudescences or relapses) is important epidemiologically for malaria elimination in Africa. Although hypnozoites will no doubt be a source, we should try to determine how frequently the origin of non-reinfection recurrences of *P. vivax* malaria involving closely related parasites may be non-circulating merozoites rather than hypnozoites.

Globally, approximately 2.5 billion people are at risk of acquiring *Plasmodium vivax* infection. Malaria caused by *P. vivax* is being diagnosed with increasing frequency in Africa.^1^ Some southern countries where it has been detected are Angola, Botswana, Mozambique, Namibia, Zambia and Zimbabwe.^1^ Knowing the parasite origin (mosquito or human tissue) of *P. vivax* infection recurrences (which can be reinfections, recrudescences or relapses) is important epidemiologically for malaria elimination in Africa. A reason is that the efficacy of drugs against parasites might vary according to their location in the body. This necessitates elucidatory research. Although hypnozoites^2^ will no doubt be a source of recurrences, we should try to determine how frequently the origin of non-reinfection recurrences of *P. vivax* malaria involving closely related parasites may be non-circulating merozoites rather than hypnozoites.

One reason why this possibility should be considered in *P. vivax* population genetics studies is the recent discovery that, in chronic infections, sequestered and multiplying extravascular asexual *P. vivax* parasites occur in vast numbers.^3,4,5,6^ Very few hepatic hypnozoites will be present and homologous recurrences can be highly suggestive of a clonal merozoite origin.^7^ That non-circulating merozoites are likely to be the source of many homologous *P. vivax* malarial recurrences is a theory I proposed in 2011 and 2012 and have advanced incrementally.^7,8,9^

As has been explained elsewhere,^10^ a few recent papers have avoided mentioning where the theory arose. This failure to acknowledge such a pertinent and unique contribution (following on from my coining of the term hypnozoite^2^ which is, unethically, poorly cited) makes those papers defective pieces of scholarship and hence non-authoritative.

This theory regarding the non-hypnozoite, intra-host parasite origin of *P. vivax* infection recurrences includes not only short-term homologous recurrences but also, for various reasons,^8,9,10^ long-term recurrences in which the parasites are likewise closely related to those from a pre-recurrence time point. The reliability of the temporal criterion that post-28-day recurrences are more likely to be relapses (these are hypnozoite-mediated) than recrudescences (which, by definition, have a merozoite origin) has been questioned.^10^ Another way to explain^8,9,10^ why some long-term homologous recurrences of *P. vivax* malaria may be recrudescences is by comparing them with long-term homologous recurrences of *Plasmodium malariae* and *Plasmodium falciparum* malaria. Those recurrences are thought to be recrudescences because a hypnozoite stage is not known to occur in the life cycle of either *P. malariae* or *P. falciparum*. There is no known reason why long-term homologous *P. vivax* malarial recurrences should not have an equivalent non-hypnozoite origin, at least sometimes.^8,9^

A drug-associated explanation for apparent relapses has also been put forward. This needs to be followed up. Recurrence patterns in groups of patients treated with the hypnozoitocide primaquine, as well as some results of mathematical modelling, have in the past been interpreted as indicating that most recurrences of *P. vivax* malaria are relapses. However, the recently elucidated mechanism of action of primaquine suggests that non-circulating merozoites in bone marrow and perhaps elsewhere too can be inactivated by the drug,^10^ in addition to hypnozoites being killed. If this is so, primaquine might not only reduce the number of subsequent relapses but also prevent an unknown percentage of recrudescences from taking place. This newly recognised possibility^10^ confuses the issue, making the parasite source of non-reinfection homologous recurrences of *P. vivax* malaria in individual cases inexplicable.

One of the most recent studies to consider the parasite origin of *P. vivax* malarial recurrences was a meta-analysis.^11^ By assuming that primaquine kills hypnozoites but not non-hypnozoite asexual stages, the authors were obliged to conclude that most of these recurrences are relapses. This may or may not be correct. At present, we simply do not know.

Understanding the parasite origin or origins of non-reinfection recurrences of *P. vivax* malaria has thus become even more difficult than it already was. Nonetheless, genotyping remains fundamental for analysing the results of drug trials and planning the control of malaria.^12^ The issues discussed above must therefore be taken into account in future molecular epidemiological research and in mathematical modelling of recurrent malaria.
